# Supplementary material for: Dynamic viability of the 2016 Mw 7.8 Kaikōura earthquake cascade on weak crustal faults
Source: Nat Commun. 2019 Mar 14;10:1213. doi: 10.1038/s41467-019-09125-w (PMC6418120; doi:10.1038/s41467-019-09125-w)
Supplement: Supplementary file 1 — Supplementary Information [file 41467_2019_9125_MOESM1_ESM.pdf]

Supplementary Table 1: Fault frictional properties assumed in this study.

Direct-effect parameter	a	0.01
Evolution-effect parameter	b	0.014
Reference slip rate	V_0	10^{-6} m/s
Steady-state low-velocity friction coefficient at slip rate V_0	f_0	0.6
Characteristic slip distance of state evolution	L	0.2 m
Weakening slip rate	V_w	0.1 m/s
Fully weakened friction coefficient	f_w	0.1
Initial slip rate	V_ini	10^{-16} m/s

Supplementary Table 2: Parameter values of the additional dynamic rupture scenarios probing the robustness of the preferred model. Variations to our preferred model are marked in bold.

	fully-weakened friction coefficient f_w	stress shape ratio ν	fluid-pressure ratio γ	unmodulated pre-stress ratio R_0	stress concentration intensity $g(0)$
preferred model	0.1	0.15	0.66	0.85	0.6
model DR1 (no deep stress concentrations)	0.1	0.15	**0.7**	**0.7**	**1**
model DR2 (increased dynamic friction)	**0.3**	**0.05**	**0.44**	0.85	0.6
model DR3 (combination of DR1 and DR2)	**0.3**	**0.05**	**0.59**	0.85	**1**

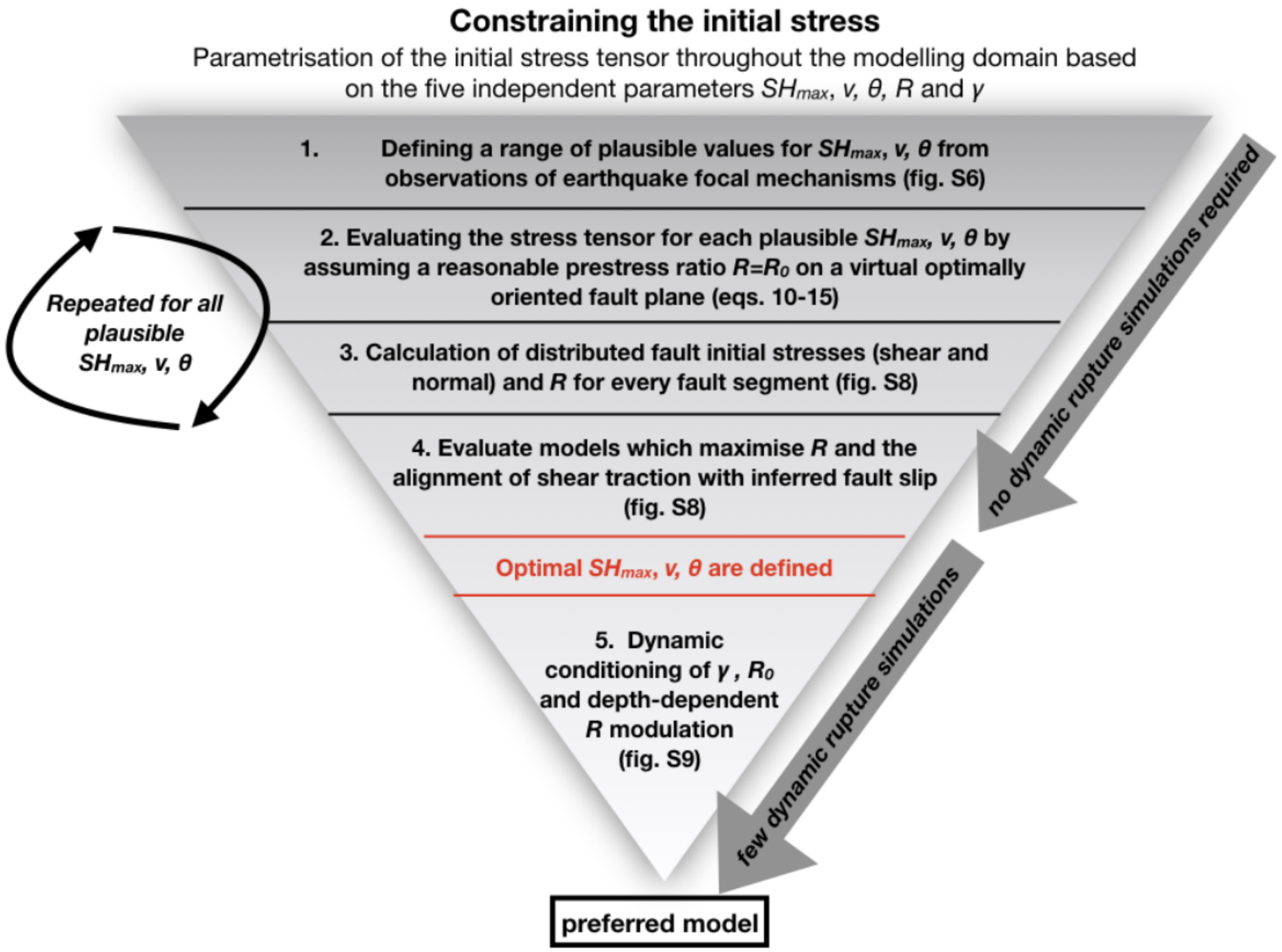

Supplementary Figure 1: Workflow for constraining the initial stress from observations and simple theoretical analysis requiring only few trial dynamic rupture simulations. The independent parameters that fully describe the initial stress tensor are: $\mathrm{SH}_{\mathrm{max}}$ denotes the azimuth of maximum horizontal compressive stress, ν is the stress shape ratio, θ is the orientation of the intermediate principal stress relative to horizontal, R is the relative prestress ratio, γ is the ratio between fluid-pressure and lithostatic confining stress, and the stress modulation functions $g(z)$ and $\Omega(z)$, all described in the text.

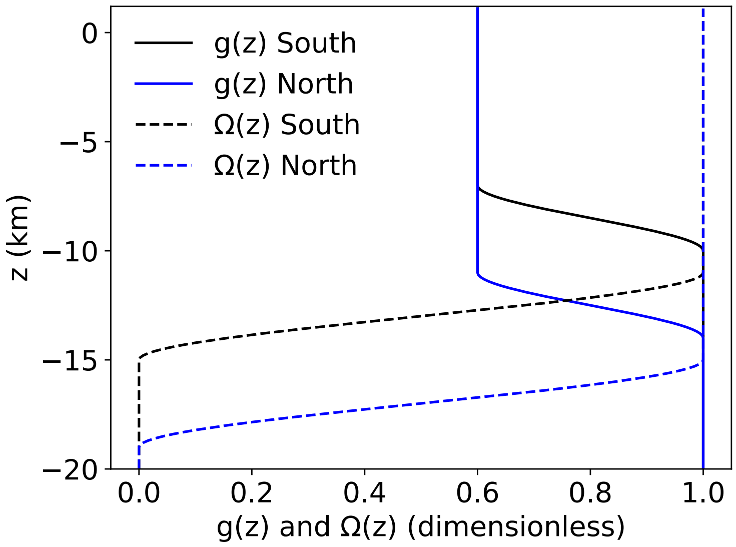

Supplementary Figure 2: Depth-dependent stress modulation functions $g(z)$ and $\Omega(z)$. The former tapers off following a Smoothstep function at some distance above the seismogenic depth z_{seis}. The latter tapers off below z_{seis}. The seismogenic depth is prescribed as slightly shallower ($z_{\mathrm{seis}} = 10.5\,\mathrm{km}$) in the Northern part of the rupture than in its Southern part ($z_{\mathrm{seis}} = 14.5\,\mathrm{km}$).

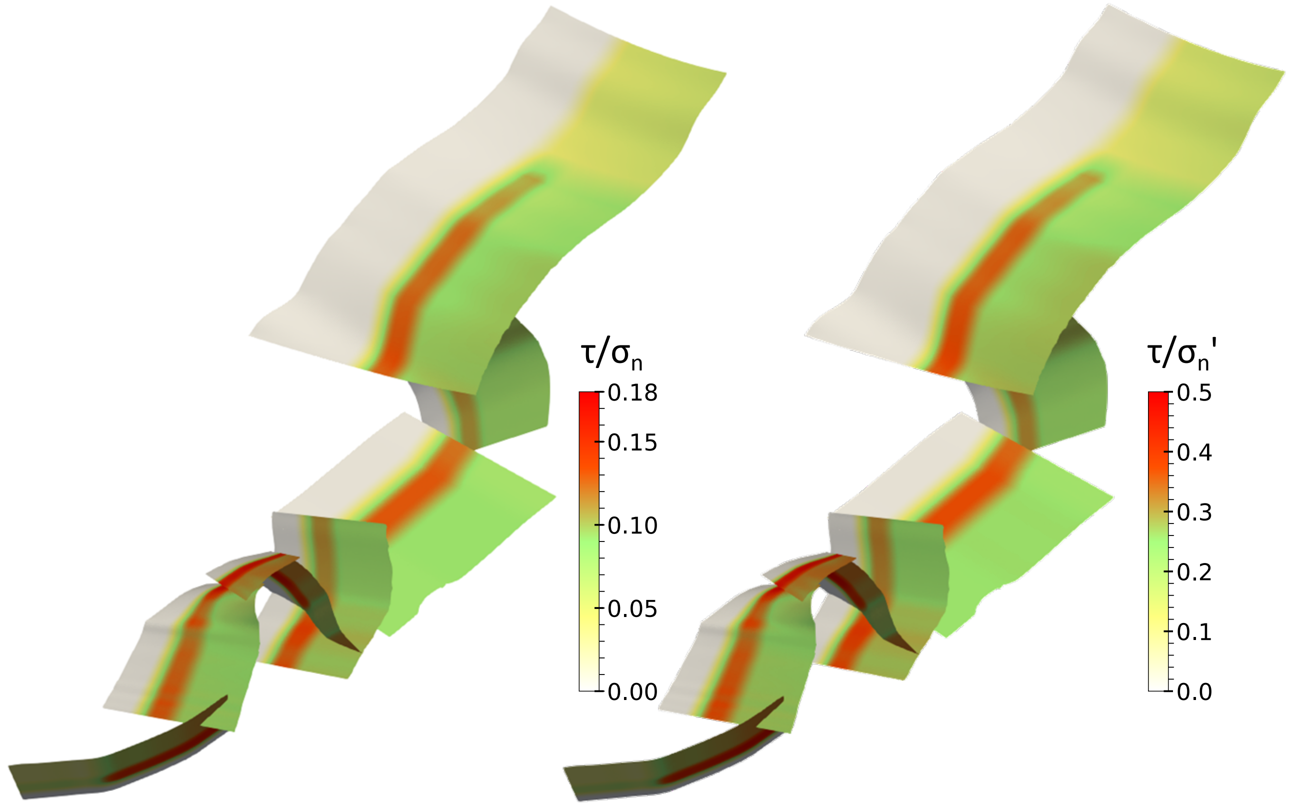

Supplementary Figure 3: Ratio of initial shear stress τ over normal stress σ_{n} (a) and over effective normal stress σ_{n}' (b).

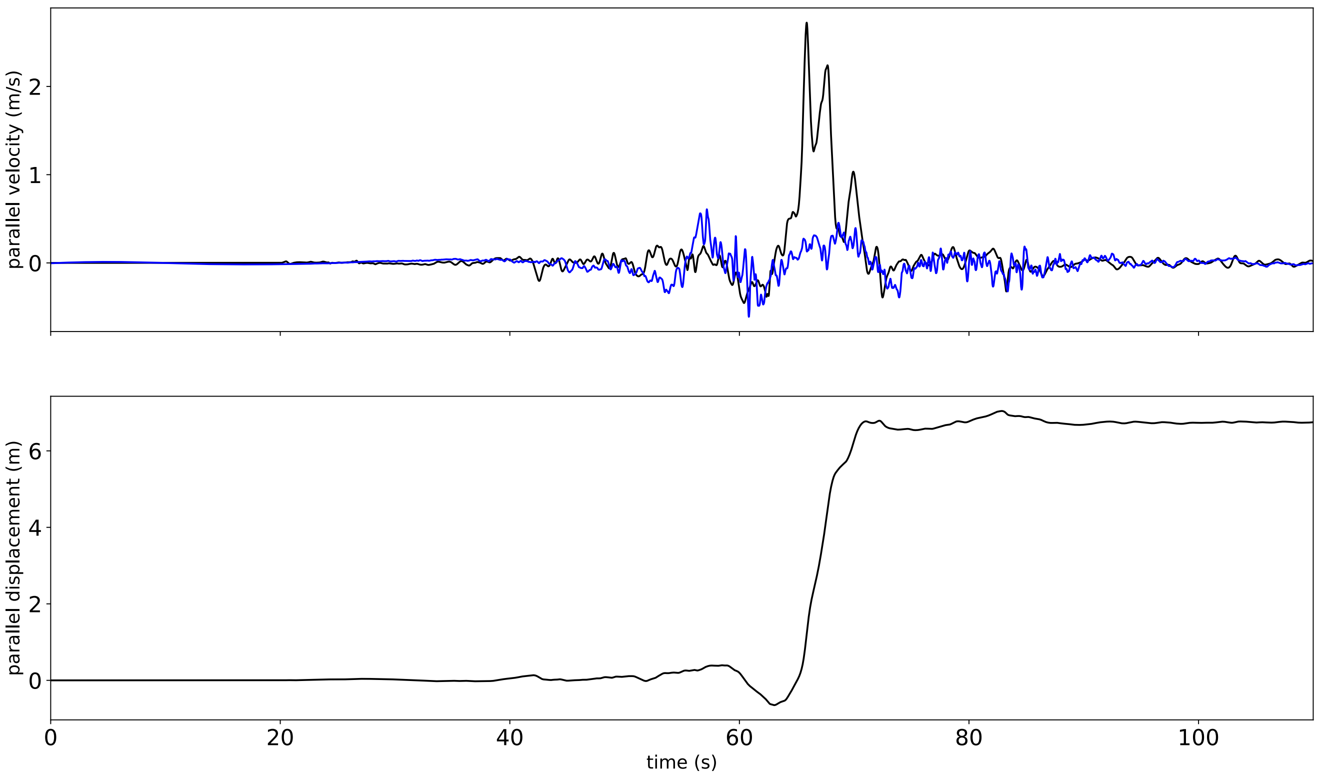

Supplementary Figure 4: Synthetic (black) and observed (blue) fault-parallel velocity and displacement waveforms at station KEKS (location shown in Supplementary Fig. 7b). The apparent slip-weakening distance D_c'' is estimated following the method of Mikumo et al.[66] as twice the fault-parallel displacement observed when the peak fault-parallel velocity is reached. We estimate $D_c'' = 5.6$m averaging over the two largest parallel velocity peaks caused by segmented on-fault dynamic rupture fronts.

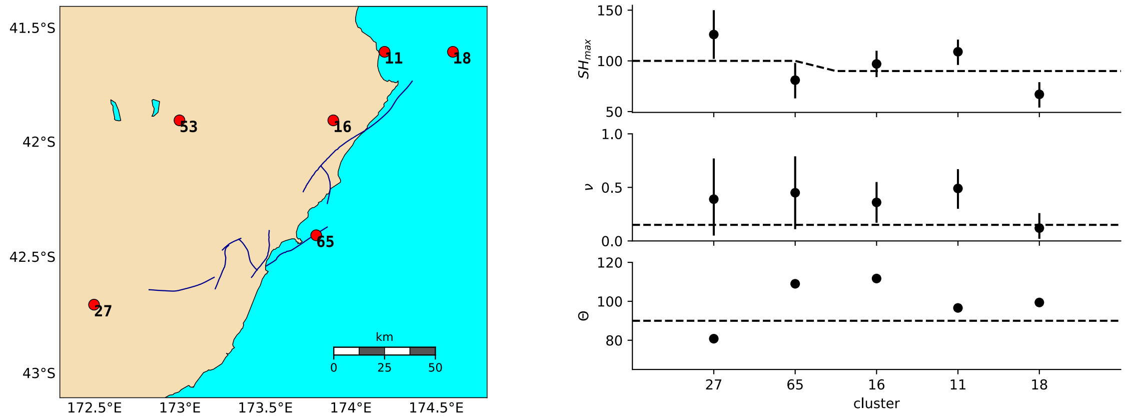

(a) (b)

Supplementary Figure 5: Observationally constrained regional stress state. (a) Centroid locations of the earthquake clusters from Townend et al.[33] that are close to the Kaikura earthquake source. We discard cluster 53 because it is too deep. (b) Stress parameters of the 5 remaining clusters. Uncertainties of SH_{max} and ν are indicated by their 10% - 90% percentile ranges (vertical bars). The dashed lines show the stress parameter values we chose.

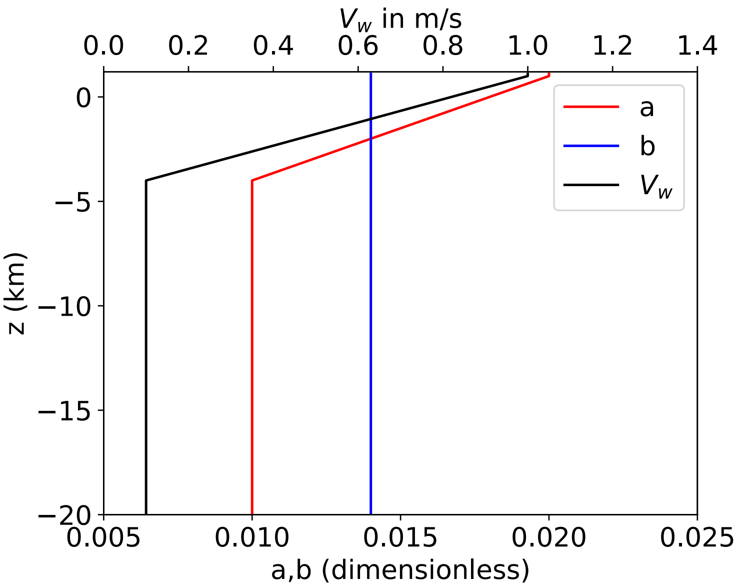

Supplementary Figure 6: Depth dependence of friction parameters.

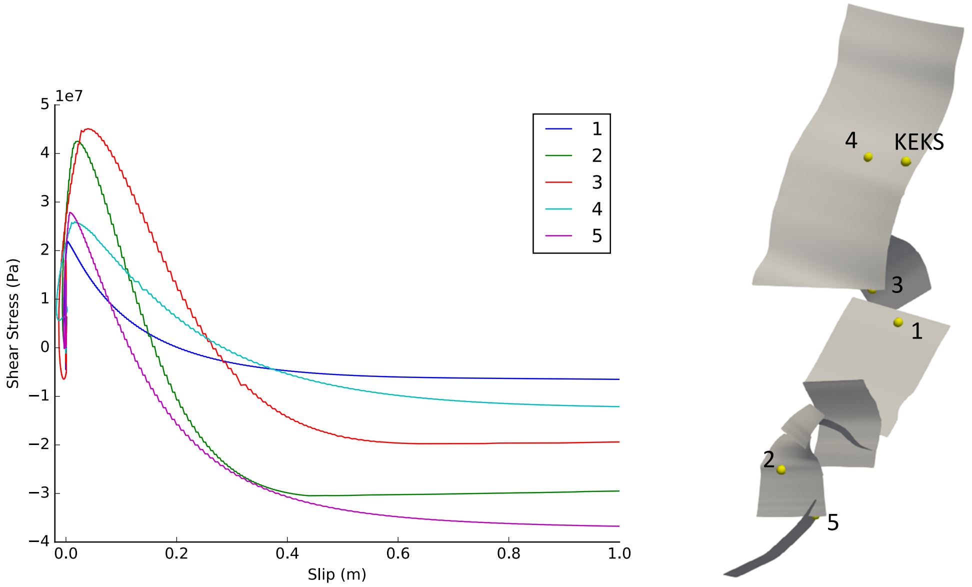

(a) (b)

Supplementary Figure 7: Slip-weakening response and equivalent critical slip-weakening distance. (a) Changes of shear traction in the direction of initial shear traction as a function of slip at 5 fault locations shown in (b). The stress drops over slip distances in the range from 0.2 to 0.5 m.

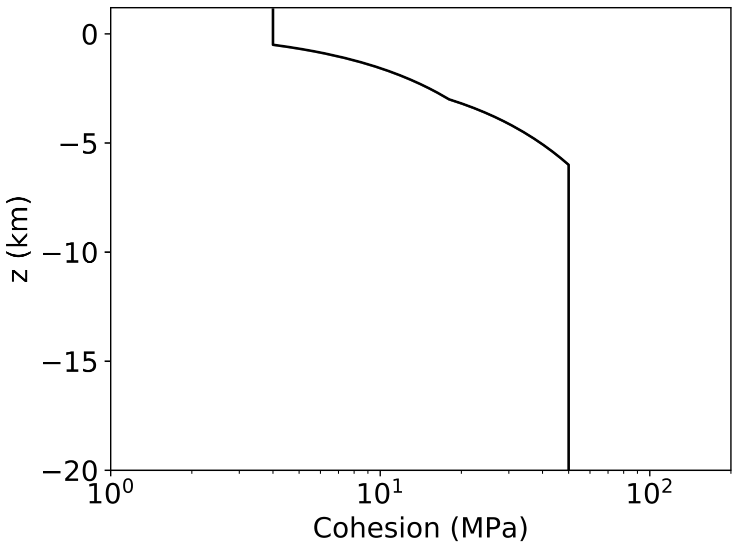

Supplementary Figure 8: Depth dependence of cohesion in the off-fault plastic yielding criterion.

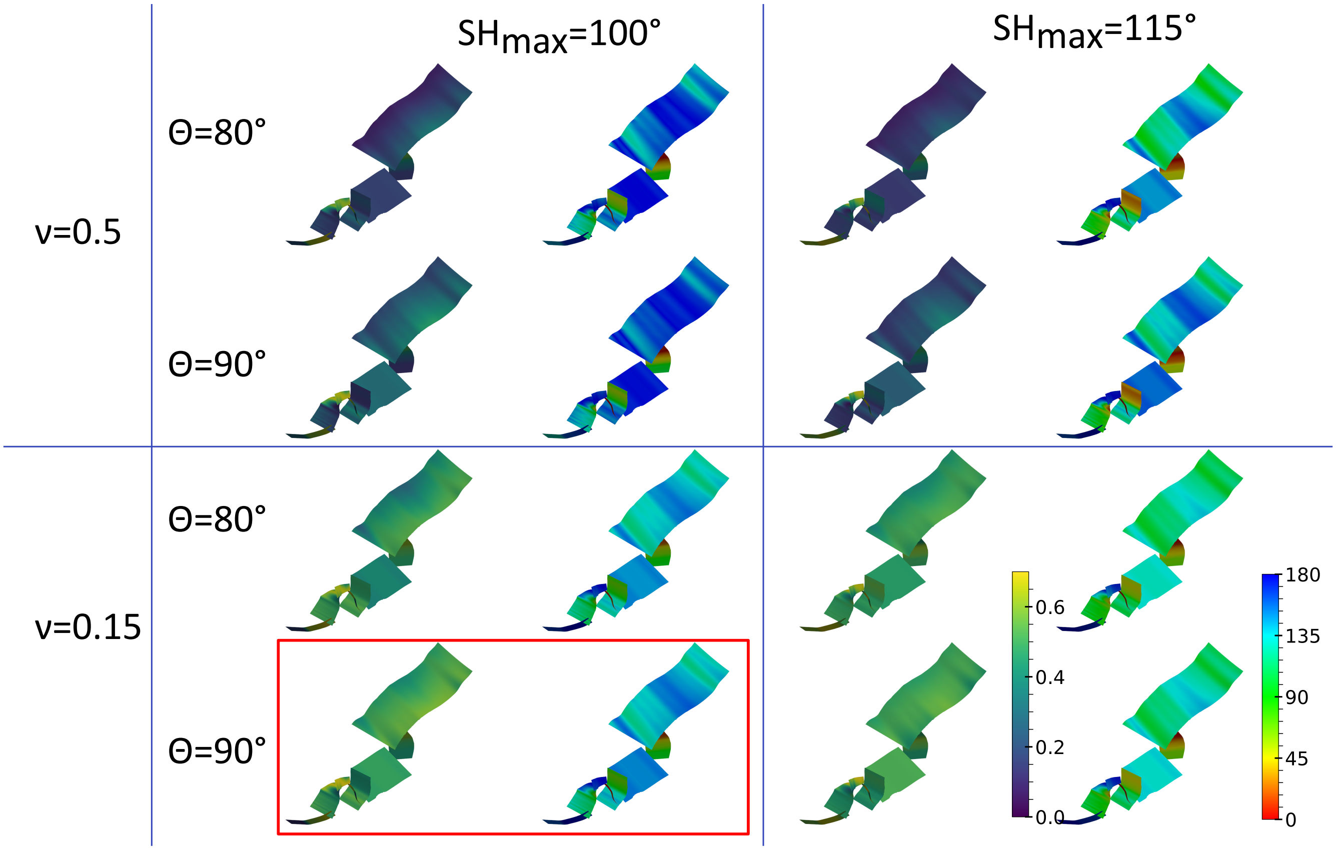

Supplementary Figure 9: A representative sample of initial stress models tested. We show 8 examples that correspond to all permutations involving the two values indicated in the labels for each stress parameter, $\mathrm{SH}_{\mathrm{max}}$, ν and θ. For each example, two plots show the spatial distribution on the fault surfaces of (left) the pre-stress ratio and (right) the rake angle of the shear traction. Here we assume a uniform $R_{\mathrm{opt}}(z) = 0.7$ on the optimal plane.

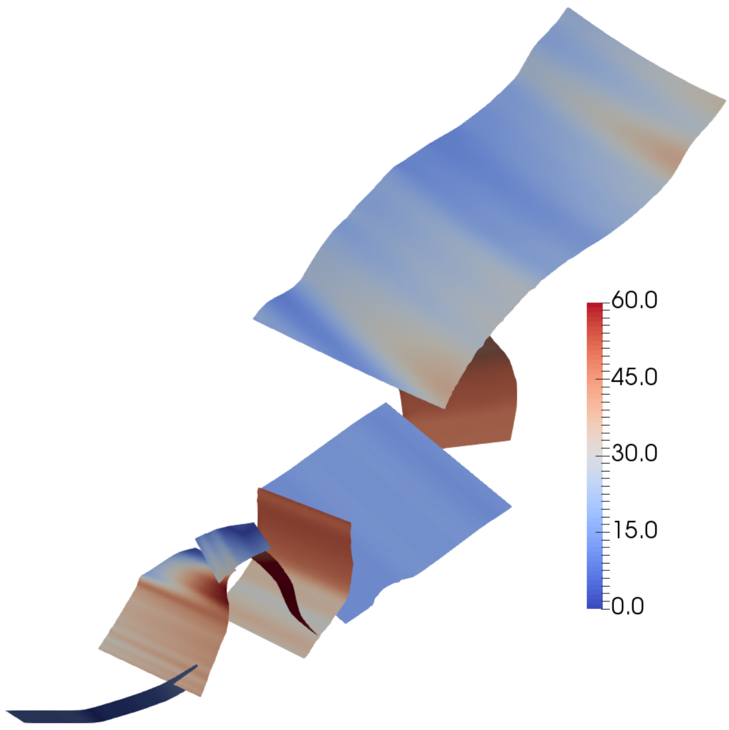

Supplementary Figure 10: Fault angle ψ relative to the maximum principal stress. Faults featuring ψ close to $\Phi = 30°$ are well oriented. To compute ψ, we first select the fault normals whose scalar product with the vector pointing toward $\mathrm{SH}_{\mathrm{max}}$ is positive. We then compute the angle ϕ between these normals and $\mathrm{SH}_{\mathrm{max}}$. Finally, we obtain ψ as $\psi = 90° - \phi$.

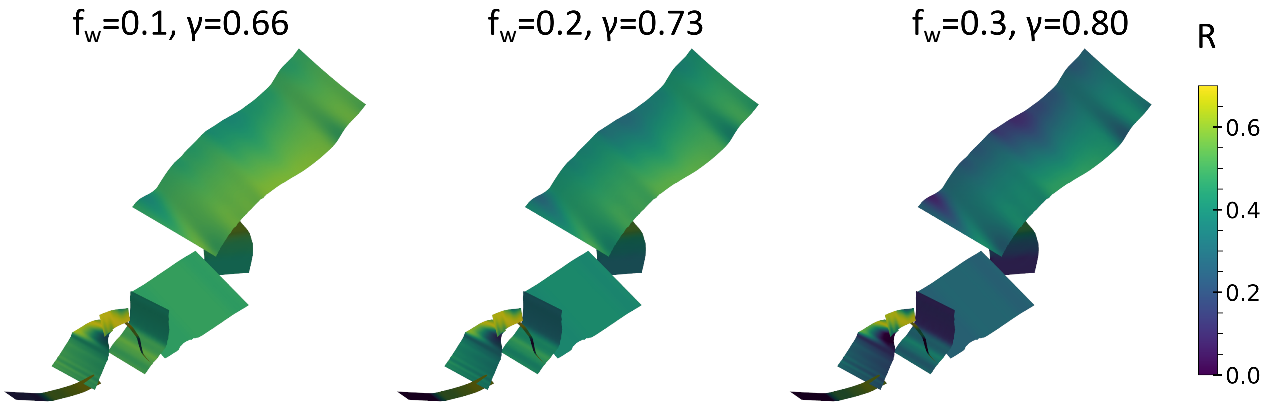

Supplementary Figure 11: Spatial distribution of the relative prestress ratio R across fault surfaces for varying values of dynamic friction coefficient assuming an intermediate stress ratio ν of 0.15 and a uniform $R_{\mathrm{opt}}(z) = 0.7$ on the optimal plane.

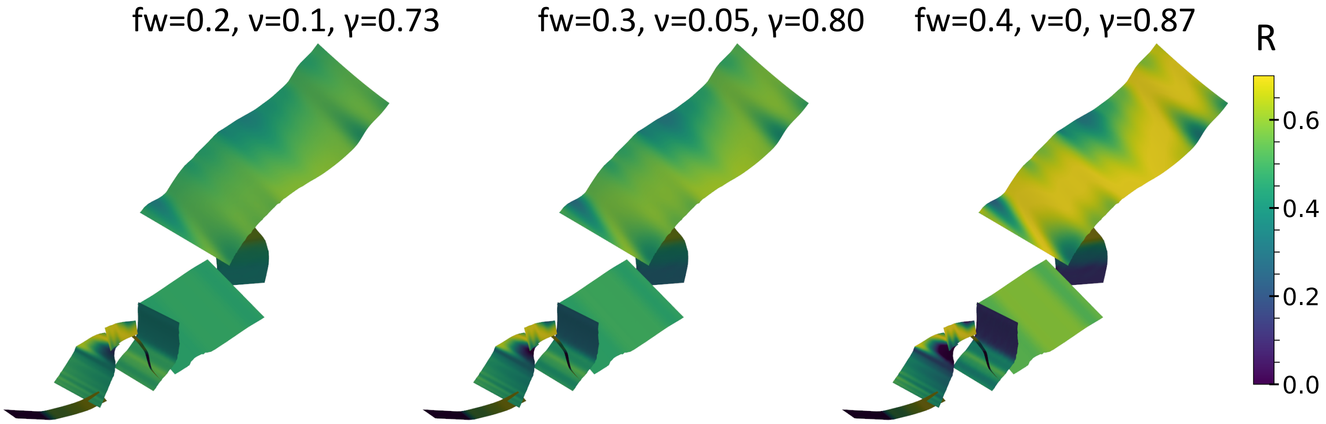

Supplementary Figure 12: Spatial distribution of the relative prestress ratio R across fault surfaces for varying values of dynamic friction coefficient assuming decreased intermediate stress ratio ν to restore the rupture potential of the dip-slip segments. The Northern part of Hundalee and the Southern part of Papatea faults experience considerably lower levels of prestress compared with the preferred model featuring $f_\mathrm{w} = 0.1$.
