# Supplementary material for: Dynamic viability of the 2016 Mw 7.8 Kaikōura earthquake cascade on weak crustal faults
Source: Nat Commun. 2019 Mar 14;10:1213. doi: 10.1038/s41467-019-09125-w (PMC6418120; doi:10.1038/s41467-019-09125-w)
Supplement: Supplementary file 2 — Description of Additional Supplementary Files [file 41467_2019_9125_MOESM2_ESM.pdf]

Description of Additional Supplementary Files

File Name: Supplementary Movie 1

Description: Absolute slip rate (m/s) across the fault network during the earthquake.

File Name: Supplementary Movie 2: Absolute slip rate (m/s) and wavefield (absolute particle velocity in m/s) across the fault network during the earthquake.

In: https://doi.org/10.5281/zenodo.2538055
